# Embedding problem solving and use of data with routine supply chain procedures: District leadership and team-based approaches improve product availability in Rwanda

**DOI:** 10.1186/2052-3211-7-S1-P11

**Published:** 2014-12-17

**Authors:** Alexis Heaton, Amanda Ombeva, Deogratias Leopold, Golbert Kazoza, Patrick Nganji, Cathy Mugeni, Megan Noel, Yasmin Chandani

**Affiliations:** 1 JSI Research & Training Institute, Inc. Washington DC, USA; 2 Rwanda Ministry of Health, Community Health Desk, Kigali, Rwanda

## Background

In Rwanda, 30,000 volunteer community health workers (CHWs) treat children under five for pneumonia, diarrhoea and malaria. A 2010 community supply chain (SC) assessment identified a lack of SC skills and poor coordination between CHWs, health centres (HCs) and districts as barriers to CHW product availability. SC4CCM tested standard resupply procedures (RSPs) and multi-level quality improvement teams (QITs) to strengthen coordination and problem-solving between levels to improve supply chain processes and outcomes.

## Method

In 2013, SC4CCM conducted a mixed-methods midline evaluation and an endline study in 2014 to understand sustainability and scalability of the QIT approach. A quantitative survey measured key supply chain indicators to compare results 12 months after launching the intervention (at midline), and another 12 months later to understand if results were sustained. Qualitative data at endline assessed enabling factors and barriers for scale up after the MOH began implementing RSPs and QITs nationally.

## Results

Midline results showed that the team-based approach led to improved outcomes. CHWs in QIT districts had 25% greater availability of the five community health products on the day of visit than the comparison group. Qualitative results confirmed the importance of multi-level teams and a structured approach in achieving results. Endline findings confirmed the role of district leadership in maintaining and scaling this intervention. While CHWs in all districts affirmed the value of the approach, establishment of QITs in new districts and continued use of data relied on leadership of HC staff, frequently predicated upon district staff engagement and participation.

## Discussion

Product availability and performance of SC tasks among CHWs can be improved by establishing multi-level teams that aid coordination and communication across levels in the health system and use data to prioritize areas for problem solving and develop local solutions. To establish and maintain meetings, leadership and on-going engagement from district staff ensures HC staff call meetings and prioritize the activities among their many other tasks. Meetings should have a known agenda, be short, and have a consistent approach to the use of data for performance monitoring and identification of problems and solutions within the team’s ability to address.

## Lessons learned

CHWs are often isolated from the mainstream health system. Strengthening their connections with HC and district staff through teams improves coordination and sets a culture of continuous improvement. Engagement by district coaches is necessary to establish QITs and ensure HCs provide the necessary leadership to sustain meetings and the approach.

